# A meta-analysis of montelukast for recurrent wheeze in preschool children

**DOI:** 10.1007/s00431-017-2936-6

**Published:** 2017-06-01

**Authors:** Hasan R. Hussein, Atul Gupta, Simon Broughton, Gary Ruiz, Nicola Brathwaite, Cara J. Bossley

**Affiliations:** 1 Faculty of Life Sciences & Medicine, Kings College London, London, UK; 2 Department of Respiratory Paediatrics, Kings College Hospital, Denmark Hill, London, SE5 9RS UK

**Keywords:** Recurrent wheeze, Meta-analysis, Montelukast, Efficacy

## Abstract

There is conflicting evidence of the effectiveness of montelukast in preschool wheeze. A recent Cochrane review focused on its use in viral-induced wheeze; however, such subgroups are unlikely to exist in real life and change with time, recently highlighted in an international consensus report. We have therefore sought to investigate the effectiveness of montelukast in all children with preschool wheeze (viral-induced and multiple-trigger wheeze). The PubMed, Cochrane Library, Ovid Medline and Ovid EMBASE were screened for randomised controlled trials (RCTs), examining the efficacy of montelukast compared with placebo in children with the recurrent preschool wheeze. The primary endpoint examined was frequency of wheezing episodes. Five trials containing 3960 patients with a preschool wheezing disorder were analysed. Meta-analyses of studies of intermittent montelukast showed no benefit in preventing episodes of wheeze (mean difference (MD) 0.07, 95% confidence interval (CI) −0.14 to 0.29; mean for montelukast 2.68 vs placebo 2.54 (*p* = 0.5)), reducing unscheduled medical attendances (MD −0.13, 95% CI −0.33 to 0.07; mean for montelukast 1.62 vs placebo 1.78 (*p* = 0.21)) and reducing oral corticosteroids (MD −0.06, 95% CI −0.16 to 0.02; mean for montelukast 0.35 vs placebo 0.36 (*p* = 0.25)). The pooled results of the continuous regimen showed no significant difference in the number of wheezing episodes between the montelukast and placebo groups (MD −0.40, 95% CI −1.00 to 0.19; mean for montelukast 2.05 vs placebo 2.37 (*p* = 0.18)).

*Conclusions*: This review highlights that the currently available evidence does not support the use of montelukast in preschool children with recurrent wheeze. We recommend further studies to investigate if a ‘montelukast responder’ phenotype exists, and how these can be easily identified in the clinical setting.

**Table:** 

**What is Known:** *• Current guidelines recommend montelukast use in preschool children with recurrent wheeze.* *• A recent Cochrane review has found montelukast to be ineffective at reducing courses of oral corticosteroids for viral-induced wheeze.*
**What is New:** *• This meta-analysis has examined all children with preschool wheeze and found that montelukast was not effective at preventing wheezing episodes or reducing unscheduled medical attendances.* *• A specific montelukast responder phenotype may exist, but such patients should be sought in larger multicentre RCTs.*

## Introduction

Wheeze is a common condition in childhood [1]; half of children experience a wheezing episode by 6 years of age [12]. Preschool wheeze is economically burdensome on healthcare [16].

There is a large heterogeneity in the manifestation and response to treatment in preschool wheeze [9]. Early studies of montelukast [11, 17] a leukotriene receptor antagonist, showed it to be effective and is widely prescribed for preschool wheeze across the globe [8]. In addition to the cost of montelukast, some children may suffer from side effects without clinical benefit.

A recent Cochrane review showed montelukast to be ineffective at reducing courses of oral corticosteroid (OCS) in viral-induced wheeze [6]. The European Respiratory Society (ERS) has recently highlighted the overlap of viral-induced and multiple-trigger wheeze [5]. This meta-analysis aims to investigate the effectiveness of montelukast in all wheezing preschool patients, which is a more clinically relevant and ‘real-life’ group.

## Methods

### Criteria for considering studies for the review

Inclusion criteria include the following:

Randomised controlled trials (RCTs) investigating the effectiveness of montelukast for recurrent wheeze in preschool children.

Children aged 6 months to 6 years with a wheezing disorder (not bronchiolitis) were included.

Children must have been randomised to receive montelukast (compared to placebo), as an intermittent (during episodes of viral upper respiratory tract infections (URTI)) or continuous therapy for 12 months. Studies had to be conducted over a 12-month period to eliminate any seasonal variation.

### Primary outcome measure

The primary outcome measure is frequency of wheezing episodes; episodes defined as symptoms treated with beta agonists.

### Secondary outcome measures

The secondary outcome measures are as follows:

1. Unscheduled medical attendance (USMA) (visiting a family doctor or trained healthcare professional or accident and emergency department or hospitalisation)
2. Number of OCS courses

### Data sources and study selection

**Fig. 1:**
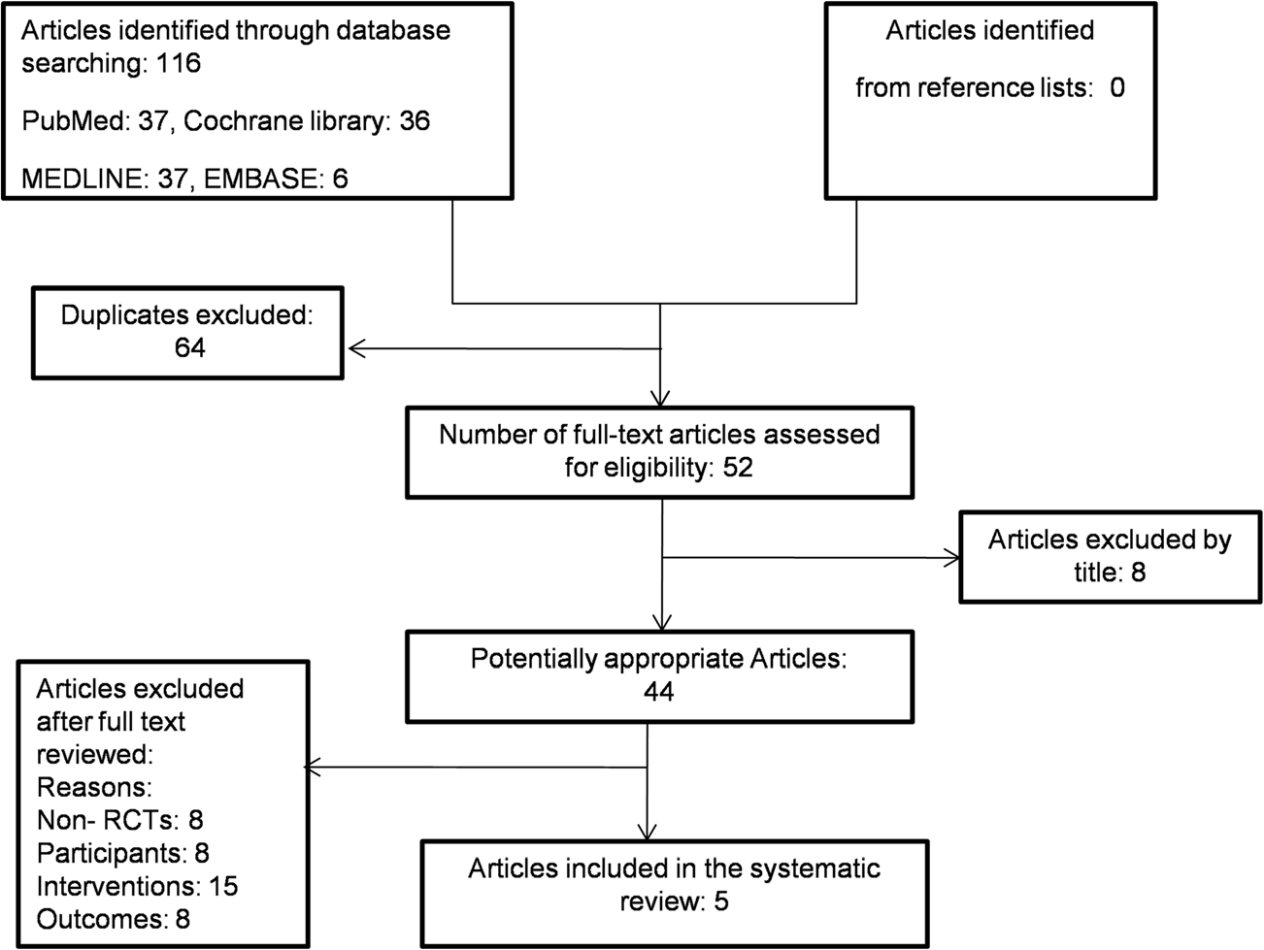
Flow chart for selection of studies

Trials were identified from Cochrane Library, PubMed, Ovid MEDLINE and Ovid EMBASE databases by two independent reviewers (HH, CB). Keywords were a combination of free texts and MeSH subject headings (Online supplement). The search strategy included filters to limit the results by the study type (RCTs only) and subject age range: infant (0–23 months) and preschool (2–5 years). No date limits were applied. No language restriction was applied. The bibliography of eligible trials was searched for relevant papers. The most recent search was conducted in April 2017. The process of study selection is shown in Fig. 1.

### Assessment of risk of bias

**Fig. 2:**
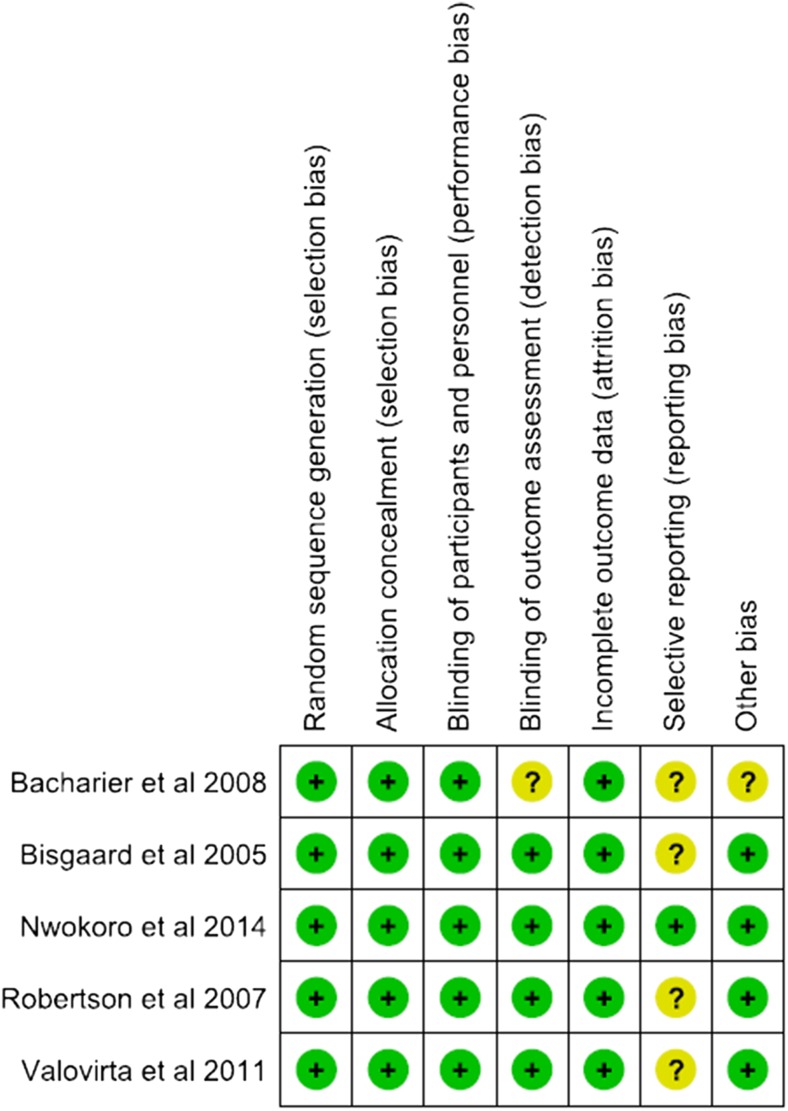
Methodological quality summary: review authors’ judgments about each methodological quality item for each included study

The risk of bias and the methodological quality of each study were assessed according to the Cochrane Collaboration’s tool. The trials have been evaluated for the presence of risk of bias in terms of allocation of randomisation sequence, concealment of allocation, blinding, handling of incomplete outcome data (online supplement), selective reporting bias and other sources of bias. The trials were of high methodological quality; therefore, the risk of bias among the studies was low. Summary assessment of the six key domains of risk of bias is presented in Fig. 2. The study by Bacharier et al. [2] was supported by the National Heart, Lung and Blood Institute (NHLBI). The WAIT study [13] was supported by the Medical Research Council and the National Institute for Health Research. Commercial sponsors provided the drugs and placebo, but all the final decisions were made by the NHLBI. Studies performed by Valovirta and Bisgaard et al. [4] were sponsored by Merck & Co. Inc. The study by Robertson et al. [15] was sponsored by Merck Sharp and Dohme (Australia) Pty. Ltd.

### Statistical analysis

Data for the selected outcomes were extracted and entered into Review Manager Software (version 5.3). Data were expressed as a weighted mean difference (MD) and 95% CI. Fixed-effect (FE) model was used to pool the data. Whenever there was a heterogeneity, random-effect (RE) model was applied. Standard deviations (SD), if they were not reported, were calculated from means and 95% CI. The study results were combined depending on the method of prescribing montelukast (intermittent or continuous). In one trial, preschool and school-age children were included [15]; we used the preschool data only.

## Results

One hundred sixteen records were identified from all databases. After completion of the study selection process, five studies (*n* = 3960) met the inclusion criteria (Table 1) [2, 4, 13, 15, 18]. All studies were conducted in high-income countries except one study [18] with centres from Africa, South America and the Middle East. Montelukast was given intermittently in four studies [2, 13, 15, 18]. Intermittent montelukast therapy was started by parents/caregivers, as a chewable tablet or oral granules (4 or 5 mg) over 12 months. In one study, in addition to episode-driven montelukast, daily montelukast therapy was also investigated [18].

**Table 1:** Characteristics of included studies

Author	N	Outcomes	Side effects	Notes/bias
Nwokoro et al. [13]	Mk, 669 PBO, 677	P: USMA S: Number and duration of WE, duration of hospital stay, number of OCS courses, time to first USMA, symptomatic days	None	● Intermittent use *● ALOX5* (5/5 and 5/x + x/x) strata ● 71.5% EVW ● 21 primary and 41 secondary care sites in the UK
Bacharier et al. [2]	MK (4 mg), 95 BIS (1 mg), 96 PBO, 47	P: Proportion of EFDs S: Symptom score, caregiver QOL, numbers and time to first OCS course, number of WEs, number of USMA, linear growth, days missed from day care and parental work	N/A	●Intermittent use ● 2:1 randomisation ● More dropouts in Mk group ● 5 clinical centres in the USA
Bisgaard et al. [4]	Mk, 278 PBO, 271	P: Number of AEE S: Number of oral and ICS courses, duration of episodes, percentage of days without asthma, severity of AEE, blood eosinophil count, proportion of patients with an AEE, time to first AEE, asthma-related resource utilisation	1 case of vomiting due to Mk overdose	● Continuous use ● Subgroup analysis based on atopic profile and blood eosinophil count ● 68 sites in 23 countries
Robertson et al. [15]	Mk, 107 PBO, 113	P: USMA S: Individual components of USMA, duration of episode, symptom score, O CS and β-agonist use, days missed from parental work and school or childcare, number of nights with disturbed sleep	None	● Intermittent use ● More children with history of atopy in Mk group ● Country: Australia
Valovirta et al. [18]	Daily Mk, 589 12-day Mk, 591 PBO, 591	P: Number of asthma episodes culminating in asthma attacks S: Symptom score, number of asthma attacks and episodes, percentage of EFDs, difference in efficacy between 2 regimens	1 case of somnolence due to Mk overdose	● Intermittent and continuous use ● Double dummy ● 111 multinational sites

All double-blind randomised placebo-controlled trial

*Mk* montelukast, *PBO* placebo, *P* primary, *S* secondary, *USMA* unscheduled medical attendance, *WE* wheezing episode, *CS* corticosteroid, *EVS* episodic viral wheeze, *MTW* multiple-trigger wheeze, *ALOX5* arachidonate 5-lipoxygenase, *BID* budesonide inhalation suspension, *EFD* episode-free day, *QOL* quality of life, *AEE* asthma exacerbation episodes, *ICS* inhaled corticosteroid

### Intermittent use of montelukast

**Fig. 3:**
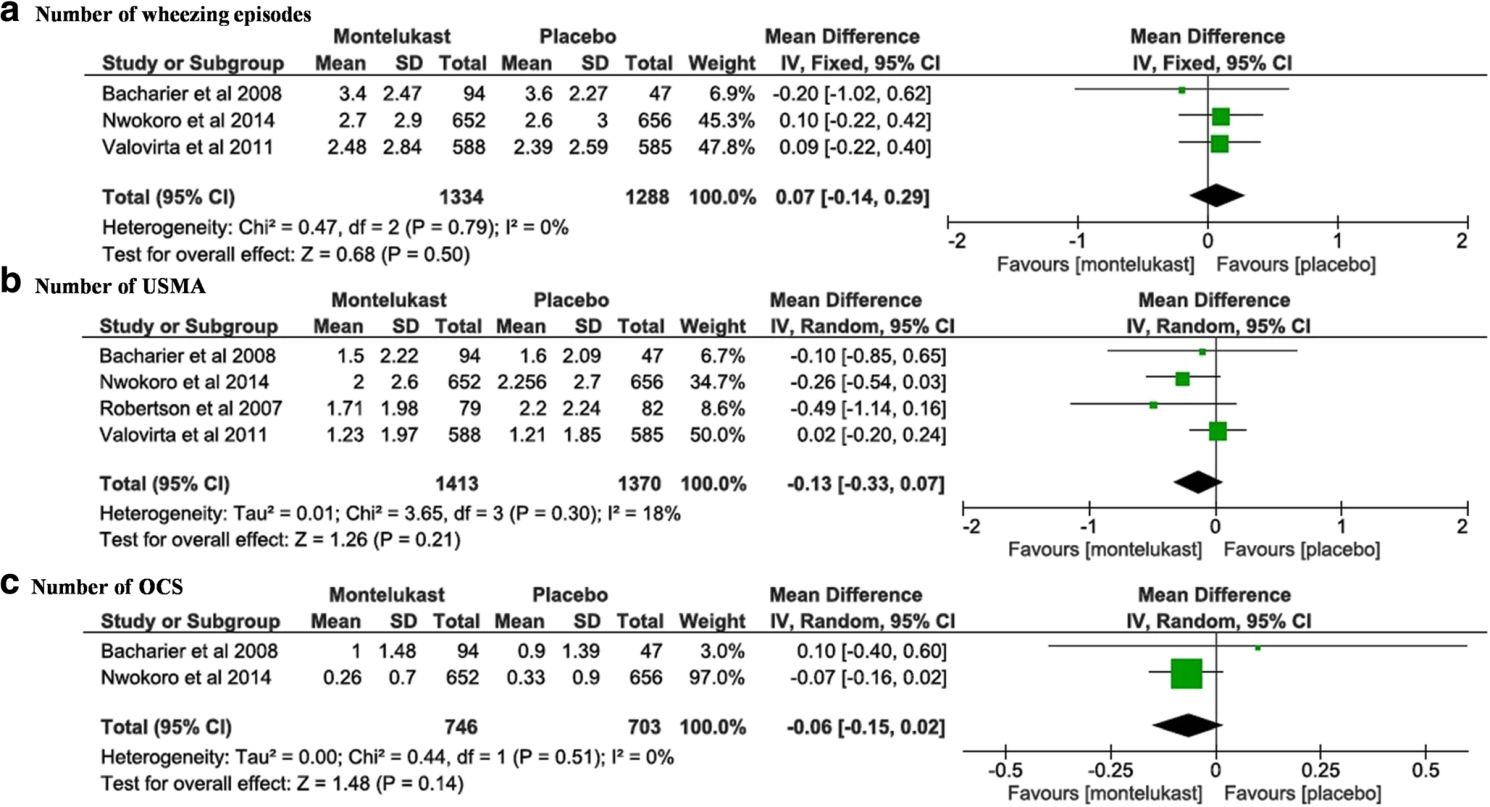
Intermittent montelukast vs placebo. **a** Numbers of wheezing episodes. **b** Unscheduled medical attendances. **c** Number of oral corticosteroid courses

The number of wheezing episodes was described in three studies [2, 13, 18]. The final study [15] reported the number of wheezing episodes whereby a child was seen by a healthcare professional. Trials showed no effects of montelukast in preventing episodes of wheeze. The pooled estimate showed no statistically significant difference (MD = 0.07, 95% CI −0.14 to 0.29, mean for montelukast 2.68 vs placebo 2.54 (*p* = 0.5)). The figure shows no heterogeneity between the studies (Fig. 3a, *p* = 0.79, *I* ^2^ statistic = 0%).

All studies which used montelukast intermittently reported USMA [2, 4, 15, 18]. The overall effect was not statistically significant (MD = −0.13, 95% CI −0.33 to 0.07, mean for montelukast 1.62 vs placebo 1.78 (*p* = 0.21)). There was a low level of heterogeneity (Fig. 3b, *p* = 0.30, *I* ^2^ statistic 18%).

The effect of montelukast on the use of OCS was described in three trials [2, 13, 15]. In one trial [15]**,** only the percentage of episodes that required OCS was presented (21% montelukast vs 24% placebo). Thus, the results could not be entered into the meta-analysis. This trial reported no significant difference between montelukast and placebo in reducing the number of OCS courses (OR, 0.80; 95% CI, 0.56 to 1.15; mean for montelukast 0.35 vs placebo 0.36 (*p* = 0.25)) [15].

The pooled estimate of the other two studies [2, 13] showed that montelukast did not significantly reduce the number of OCS courses (MD = −0.06, 95% CI −0.15 to 0.02, *p* = 0.14). There was no heterogeneity between the trials (Fig. 3c, *p* = 0.51, *I* ^2^ statistic 0.0%).

### Continuous use of montelukast

**Fig. 4:**
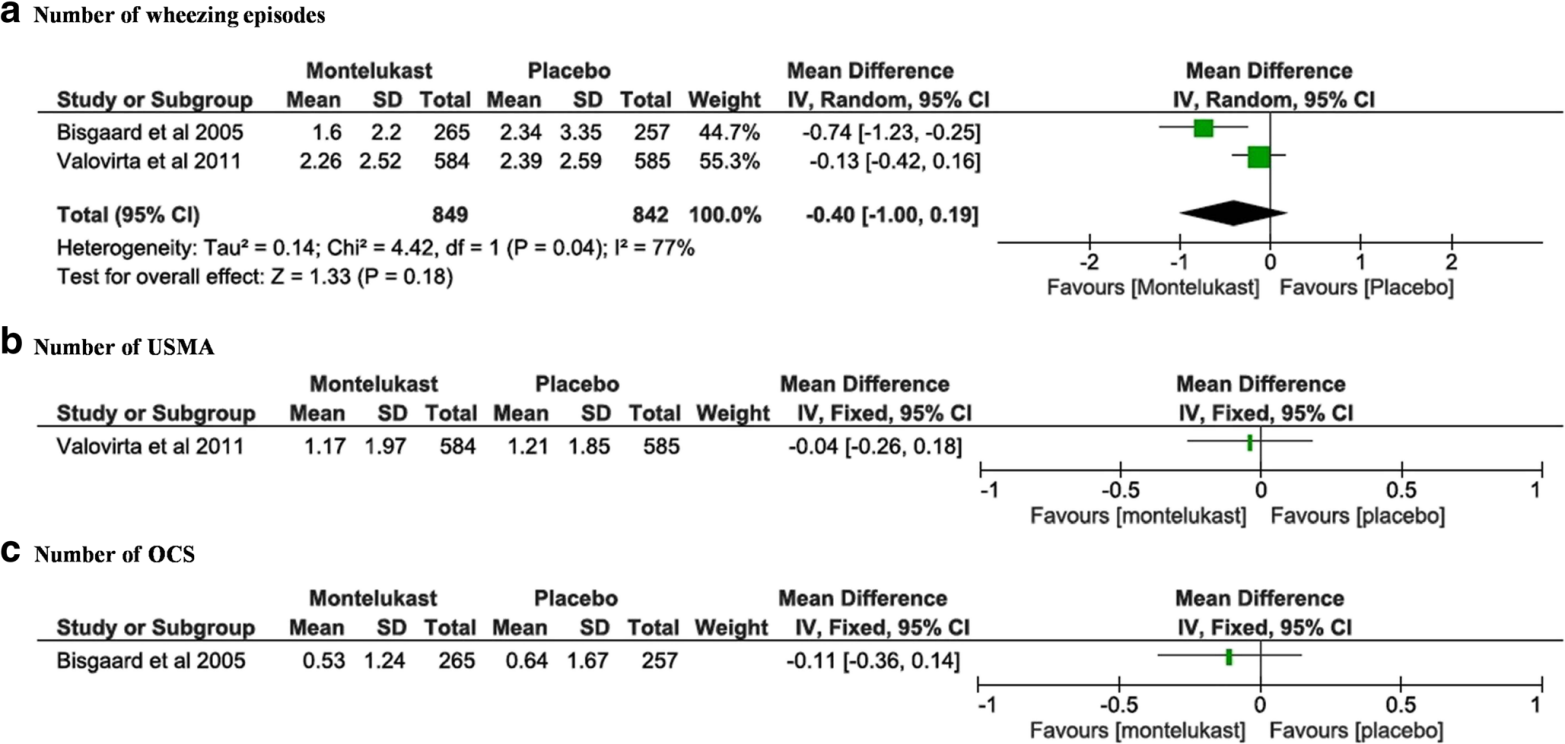
Continuous montelukast vs placebo. **a** Number of wheezing episodes. **b** Unscheduled medical attendances [18]. **c** Number of oral corticosteroid courses [4]

Only 2 of the 5 included studies investigated regular continuous montelukast (*n* = 1691) [4, 18]. The pooled estimate comparing the number of wheezing episodes was not statistically significant (MD = −0.40, 95% CI −1.00 to 0.19, mean for montelukast 2.05 vs placebo 2.37 (*p* = 0.18)); analysis showed that there was a substantial heterogeneity between the included studies (Fig. 4a, *p* = 0.04, *I* ^2^ statistic = 77%).

One study [18] described no statistically significant difference in the number of USMA between montelukast and placebo (Fig. 4b, MD = −0.04, 95% CI −0.26 to 0.18). The other [4] showed no statistical significance in the number of patients presenting with at least one USMA (MD = −0.11, 95% CI −0.36 to 0.14). As outcome measures differed, the data could not be pooled.

The number of OCS courses was reported in the study of Bisgaard et al. only [4]. It showed no statistically significant difference between montelukast and placebo in reducing the number of OCS (Fig. 4c, MD = −0.11, 95% CI −0.36 to 0.14).

### Adverse events

There was no significant difference in the incidence of adverse events between the placebo and montelukast. Two participants were suffered from somnolence and vomiting due to the montelukast overdose [4, 18]. The trials indicated that adverse events related to the intervention rare and montelukast was safe in preschool children.

## Discussion

This systematic review of pooled data shows no evidence of benefit of the use of intermittent or continuous montelukast on the number of wheezing episodes, USMA or OCS use for recurrent wheeze in preschool children. However, there may be subgroups of children with preschool wheeze who do respond to montelukast, but there were insufficient data to determine specific phenotypes of responders, apart from one study which did suggest a genotype which was linked with greater response.

The clinical question is an important one. The use of montelukast in preschool wheeze is recommended in the British Thoracic Society (BTS) guidelines [7]; it is widely used across the world and should only be recommended if it is actually helpful. The added value of this study compared to the recent Cochrane review [6] is the inclusion of all types of preschool wheeze; therefore, more akin to real life, it therefore makes the clinical question more relevant and clinically applicable. Our primary outcome measure was also very clinically relevant, being number of wheezy episodes.

The recent Cochrane review [6] used courses of OCS as a primary outcome measure; however, evidence points to the ineffectiveness of OCS in preschool wheeze. Therefore, by using this as an outcome measure, subtle changes in outcome may not be picked up, due to inconsistencies of prescribing across healthcare professionals. The use of corticosteroids in this group of children may vary from clinician to clinician, whereas the frequency of wheezing episodes is more accurate and clinically applicable.

One of the disadvantages of our analysis was that there was some heterogeneity in the outcome set in the studies chosen for review; thus, some outcomes which we did not select were unable to be included within the pooled analysis. For example, in the study by Bisgaard et al. [4], we could not compare USMAs, because in this study, they compared the proportion of patients who had at least one USMA rather than the number of unscheduled visits (37% for montelukast; 42% for placebo).

A further disadvantage was that although the entry criteria of the primary studies were generally similar, there were some differences in the baseline characteristics of the populations of the studies. Analysis showed a significant heterogeneity between the two studies used in the meta-analysis of those given continuous montelukast (*p* = 0.04, *I* ^2^ statistic = 77%). The study performed by Bisgaard et al. [4] included patients with quite mild symptoms of asthma, compared to those included in the study by Valovirta et al. [18]. In the latter study, the patients had moderate-severe asthma (intermittent symptoms as well as one course of OCS or hospitalisation in the previous year) [18]. There was another study which analysed relatively mild asthma [15].

The fact that our meta-analysis looked at preschool wheeze altogether, without separation for viral-induced or multiple-trigger wheeze, is a great strength of the current analysis compared to other meta-analyses [6]. A recent international consensus report disputes the use of such terms in preschool wheeze [5], as it seems that there is huge overlap between these phenotypes as well as change over time; thus, separating patients in this way may not be clinically relevant. This makes our study more clinically applicable to the general population.

A further advantage was the strict criteria for selection meant that only those studies with the highest quality were included, and there was subsequently little bias or doubt of the validity of the data. However, with such stringent criteria set, some studies were excluded which may have been useful. This includes a number of studies which did not span 12 months. The reason for excluding such studies was to eliminate any seasonal variation. One excluded large multicentre multinational RCT [11] which enrolled over 600 children, given montelukast or placebo for 12 weeks, showed improvement in episode-free days, symptoms, use of OCS, β-agonist use and serum eosinophil counts. The population studied seemed to be >50% atopic and many suffered from daily symptoms, maybe more akin to the previous term of multiple-trigger wheeze. It is important, however, to note that this particular study was conducted by the pharmaceutical company marketing montelukast.

In all primary studies, recording of the symptoms and initiating of the intervention in the intermittent montelukast studies were carried out by parents/caregivers. Although in all the trials, the parents were contacted either through telephone or visits, only one study provided an educational program to the parents on recognising symptoms which were more likely to represent respiratory tract infection and followed by wheezing [2]. It is possible that the initiation of treatment was too long after the onset of symptoms, causing stimulation of the immune response by the virus and thus failure of the montelukast therapy [2].

Some of the trials included children aged 6–24 months, which could incorporate some patients with post bronchiolitis wheeze [2, 13, 18]. This may have led to negative findings, because a Cochrane review reported that montelukast was not effective in reducing the incidence of recurrent wheezing, symptom-free days or relevant usage of corticosteroid in patients with post-bronchiolitis wheezing [14]. Interestingly, two included trials that excluded children younger than 2 years showed significant improvements in montelukast group compared with placebo group [4, 15]. Subgroup analysis showed better outcomes for children 2 years of age and older (*p* = 0.017) [18]. It is thus possible that there may have been some exaggeration of negative results due to the inclusion of children less than 2 years of age.

We wanted to perform subgroup analysis to check which patients were ‘montelukast responders’, especially checking if serum eosinophil or atopy predicted response. Unfortunately, there were insufficient data available to analyse this. Nwokoro et al. [13] did perform subgroup analysis and found that those patients with ALOX5 5/5 genotype had less USMA on montelukast compared with placebo, but there was some overlap between the groups. Repeatability in further large RCTs of such results would need to be performed for this to be convincing.

Since this meta-analysis has been performed, a further meta-analysis has been performed comparing the effectiveness of continuous and intermittent high-dose inhaled corticosteroid (ICS), with placebo and montelukast at preventing a severe exacerbation in preschool wheeze [10]. Continuous ICS and intermittent ICS were both found to be effective but were also found to be significantly more effective than montelukast at preventing a severe attack [10]. This meta-analysis illustrated the strong and consistent evidence that ICS is effective in preschool wheeze. This paper, as well as our findings, may influence a change in protocol for the treatment of preschool wheeze. There have also been some early data to suggest that azithromycin therapy in severe preschool wheeze may prevent a severe exacerbation [3, 19], but more work in this area is required, due to the increase in bacterial resistance following such medication.

This meta-analysis shows that, compared with placebo, 12 months of intermittent or continuous montelukast was not associated with significant reduction in the frequency of wheezing episodes, USMA or need for OCS use. This may call into question the BTS [7] recommendations for the use montelukast in preschool wheeze.

## Recommendations

No benefit was seen with montelukast for preschool wheeze from the limited well-conducted RCTs over at least 12 months in preschool children with recurrent wheeze. Future trials should be adequately powered for the predefined subgroup analysis to identify the subgroup of children most likely to exhibit a beneficial treatment response to montelukast.

## References

[1] Bacharier LB (2010). Viral-induced wheezing episodes in preschool children: approaches to therapy. Curr Opin Pulm Med.

[2] Bacharier LB, Phillips BR, Zeiger RS, Szefler SJ, Martinez FD, Lemanske RF, Sorkness CA, Bloomberg GR, Morgan WJ, Paul IM (2008). Episodic use of an inhaled corticosteroid or leukotriene receptor antagonist in preschool children with moderate-to-severe intermittent wheezing. J Allergy Clin Immunol.

[3] Bacharier LB, Guilbert TW, Mauger DT, Boehmer S, Beigelman A, Fitzpatrick AM (2015). Early administration of azithromycin and prevention of severe lower respiratory tract illnesses in preschool children with a history of such illnesses: a randomized clinical trial. JAMA.

[4] Bisgaard H, Zielen S, Garcia-Garcia ML, Johnston SL, Gilles L, Menten J, Tozzi CA, Polos P (2005). Montelukast reduces asthma exacerbations in 2- to 5-year-old children with intermittent asthma. Am J Respir Crit Care Med.

[5] Brand PL, Caudri D, Eber E, Gaillard EA, Garcia-Marcos L, Hedlin G, Henderson J, Kuehni CE, Merkus PJ, Pedersen S (2014). Classification and pharmacological treatment of preschool wheezing: changes since 2008. Eur Respir J.

[6] Brodlie MGA, Rodriguez-Martinez CE, Castro-Rodriguez JA, Ducharme FM, McKean MC (2015) Leukotriene receptor antagonists as maintenance and intermittent therapy for episodic viral wheeze in children. Cochrane Database Syst Rev, **Issue 10 Art No: CD008202**. doi:10.1002/14651858CD008202pub2 10.1002/14651858.CD008202.pub2 PMC6986470 26482324

[7] BTS/SIGN (2016) British guideline on the management of asthma. pp 64–79. https://www.brit-thoracic.org.uk/document-library/clinical-information/asthma/btssign-asthma-guideline-2016/

[8] Bush A (2015). Montelukast in paediatric asthma: where we are now and what still needs to be done?. Paediatr Respir Rev.

[9] Hon KLE, Leung TF, Leung AK (2014). Clinical effectiveness and safety of montelukast in asthma. What are the conclusions from clinical trials and meta-analyses?. Drug Des Devel Ther.

[10] Kaiser SV, Huynh T, Bacharier LB, Rosenthal JL, Bakel LA, Parkin PC, Cabana MD (2016) Preventing exacerbations in preschoolers with recurrent wheeze: a meta-analysis. Pediatrics 137(6):pii:e20154496. doi:10.1542/peds.2015-4496 10.1542/peds.2015-4496 27230765

[11] Knorr B, Franchi LM, Bisgaard H, Vermeulen JH, LeSouef P, Santanello N, Michele TM, Reiss TF, Nguyen HH, Bratton DL (2001). Montelukast, a leukotriene receptor antagonist, for the treatment of persistent asthma in children aged 2 to 5 years. Pediatrics.

[12] Martinez FD, Wright AL, Taussig LM, Holberg CJ, Halonen M, Morgan WJ (1995). Asthma and wheezing in the first six years of life. N Engl J Med.

[13] Nwokoro C, Pandya H, Turner S, Eldridge S, Griffiths CJ, Vulliamy T, Price D, Sanak M, Holloway JW, Brugha R (2014). Intermittent montelukast in children aged 10 months to 5 years with wheeze (WAIT trial): a multicentre, randomised, placebo-controlled trial. Lancet Respir Med.

[14] Peng W-S, Chen X, Yang X-Y, Liu E-M (2014). Systematic review of montelukast’s efficacy for preventing post-bronchiolitis wheezing. Pediatr Allergy Immunol.

[15] Robertson CF, Price D, Henry R, Mellis C, Glasgow N, Fitzgerald D, Lee AJ, Turner J, Sant M (2007). Short-course montelukast for intermittent asthma in children: a randomized controlled trial. Am J Respir Crit Care Med.

[16] Stevens C, Turner D, Kuehni C, Couriel J, Silverman M (2003). The economic impact of preschool asthma and wheeze. Eur Respir J.

[17] Straub DA, Moeller A, Minocchieri S, Hamacher J, Sennhauser FH, Hall GL, Wildhaber JH (2005). The effect of montelukast on lung function and exhaled nitric oxide in infants with early childhood asthma. Eur Respir J.

[18] Valovirta E, Boza ML, Robertson CF, Verbruggen N, Smugar SS, Nelsen LM, Knorr BA, Reiss TF, Philip G, Gurner DM (2011). Intermittent or daily montelukast versus placebo for episodic asthma in children. Ann Allergy Asthma Immunol.

[19] Wise J (2015). Early use of azithromycin may reduce severity of wheezing, study finds. BMJ.

